# Antioxidant Effect of Tyr-Ala Extracted from Zein on INS-1 Cells and Type 2 Diabetes High-Fat-Diet-Induced Mice

**DOI:** 10.3390/antiox11061111

**Published:** 2022-06-02

**Authors:** Jinghui Zhai, Yuhua Zhu, Yi Wu, Na Li, Yue Cao, Yi Guo, Li Xu

**Affiliations:** 1 Key Laboratory for Molecular Enzymology and Engineering, The Ministry of Education, National Engineering Laboratory for AIDS Vaccine, School of Life Sciences, Jilin University, Changchun 130012, China; zhaijh@jlu.edu.cn (J.Z.); zhuyh18@mails.jlu.edu.cn (Y.Z.); alee1019@yeah.net (N.L.); yue.cao@ciac.ac.cn (Y.C.); 2 Department of Pharmacy, First Hospital of Jilin University, Changchun 130021, China; 3 College of Pharmacy, Jilin University, Changchun 130033, China; wuyi@jlu.edu.cn

**Keywords:** type 2 diabetes, tyr-ala, INS-1 cells, peptide, reactive oxygen species

## Abstract

Type 2 diabetes mellitus (T2DM) is associated with an oxidative milieu that often leads to adverse health problems. Bioactive peptides of zein possess outstanding antioxidant activity; however, their effects on hyperglycemia-related oxidative stress remain elusive. In the present study, the dipeptide Tyr-Ala (YA), a functional peptide with typical health benefits, was applied to alleviate oxidative stress in pancreatic islets under hyperglycemic conditions. By detecting viability, antioxidant ability, and insulin secretion in INS-1 cells, YA showed excellent protection of INS-1 cells from H_2_O_2_ oxidative stress, erasing reactive oxygen species (ROS) and promoting insulin secretion. Moreover, by Western blotting, we found that YA can regulate the PI3K/Akt signaling pathway associated with glycometabolism. After establishing a T2DM mice model, we treated mice with YA and measured glucose, insulin, hemoglobin A1C (HbA1c), total cholesterol (TC), triglyceride (TG), and malonaldehyde (MDA) levels and activities of superoxide dismutase (SOD) and glutathione (GSH) from blood samples. We observed that YA could reduce the production of glucose, insulin, HbA1c, TC, TG, and MDA, in addition to enhancing the activities of SOD and GSH. YA could also repair the function of the kidneys and pancreas of T2DM mice. Along with the decline in fasting blood glucose, the oxidative stress in islets was alleviated in T2DM mice after YA administration. This may improve the health situation of diabetic patients in the future.

## 1. Introduction

Diabetes mellitus (DM) is a complex glucolipid metabolism disorder closely related to the environment and genetics [1]. Ninety to ninety-five percent of DM patients have type II diabetes mellitus (T2DM), which is characterized by chronically elevated levels of glucose in the blood [2]. The increasing prevalence of T2DM, with a high mortality rate worldwide, is a crucial health problem [3]. As many factors cause T2DM, its pathogenesis remains unclear [4]. Insulin resistance (IR) and islet β-cell failure are the key pathogenic factors of T2DM [5].

IR is always accompanied by the occurrence and development of T2DM, while islet β-cell failure is a necessary component of T2DM. With the development of IR, insulin signaling is altered, leading to reduced glucose uptake by muscle, liver, and adipose cells, elevating blood glucose and resulting in T2DM [6]. Islet β-cell failure is caused by mitochondrial oxidative elevated glucose metabolism and increased free fatty acids (FFAs), which increase the mitochondrial membrane potential (MMP) and the production of peroxides [7]. The generation of reactive oxygen species (ROS) by changing the redox state of cells plays an important role in regulating metabolism. ROS play an important role in β-cell failure when blood glucose levels are high [8,9]. Under in vitro conditions, however, it is difficult to simulate the long-term excessive energy situation, excessive burden on β-cells, and mitochondrial function defects. Therefore, the production of ROS in β-cells, which leads to oxidative damage and apoptosis, can be used as a model of β-cell failure [10]. The triglyceride (TG)/FFA cycle is likely to protect β-cells by avoiding excessive fuel, increased mitochondrial membrane potential, and ROS production. The use of the TG/FFA cycle to avoid β-cell damage plays a key role in β-cell compensatory processes [11]. This information can be used as a new direction for further studies on islet cell biology. Human amyloid (hA) is a 37-amino acid peptide hormone linked by a disulfide bond between amino acids 2 and 7. Several studies have shown that amyloid fibers are cytotoxic, and islet amyloid peptide oligomers can cause cell membrane instability and induce oxidative stress and mitochondrial damage in the cell [12,13]. Eventually, this leads to β-cell apoptosis and damages islet function. This is one of the most important pathogenic factors in T2DM [14].

The relief of oxidative stress mainly depends on various antioxidants inhibiting the excessive production of ROS. Some bioactive substances extracted from natural products have received increasing attention due to their excellent antioxidant capacity [15,16]. Zein peptide, a small molecule peptide, has various biological activities, such as antioxidant and anti-inflammation [17,18]; however, the molecular mechanism involved is still unclear. Tyr-Ala (YA) of zein was separated using protease hydrolysis, ultrafiltration, gel column chromatography, and reversed-phase high-performance liquid chromatography by our group [19]. The YA with 1,1-diphenyl-2-picrylhydrazyl free radical quenching ability, 2,2′-azino-bis (3-ethylbenzothiazoline-6-sulfonic acid) radical quenching activity, and superoxide anion quenching ability may play an important role in regulating blood glucose levels; hence, we investigated the effect of YA on T2DM.

**Figure 1:**
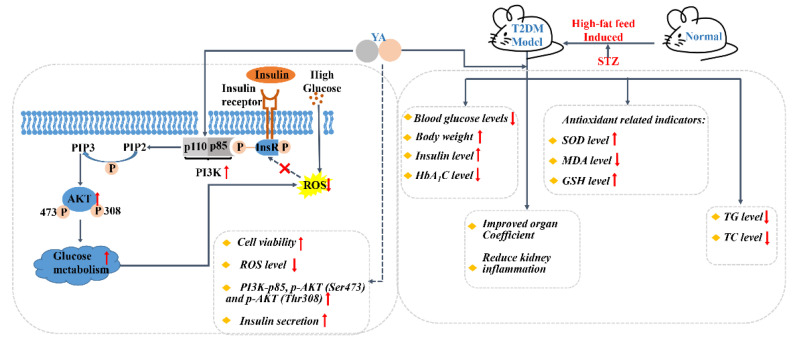
Schematic experiments of the YA effect on diabetes in vitro and in vivo. In cell assays, YA treatment increased cell viability and decreased intracellular ROS accumulation and increased insulin secretion in INS-1 cells exposed to H_2_O_2_, human amylin, or high glucose stimulation, which was achieved by activating the PI3K/AKT signaling pathway. In in vivo experiments, T2DM model was established by feeding a high-fat diet daily for 10 weeks and injecting STZ for 3 days in the fourth week. In T2DM mice, YA administration for 6 weeks improved the mice’s health, increased insulin secretion levels, and decreased blood sugar levels. It also increased the expression of antioxidant enzymes and decreased the levels of TG and TC. (ROS: reactive oxygen species; InsR: insulin receptor; PIP2: phosphatidylinositol 4, 5-bisphosphate; PIP3: phosphatidylinositol-3, 4, 5-triphosphate; AKT: protein kinase A; PI3K: Phosphatidylinositol 3-kinase; YA: Tyr-Ala; STZ: streptozocin).

In our study, in vitro, the INS-1 cell line was used to explore the molecular mechanism of YA against oxidative stress by H_2_O_2_ and human amylin, or high glucose stimulation. In vivo, high-fat diet-induced diabetic mice were used as the animal model to further estimate the antioxidant capacity of YA in T2DM, which is relevant for assessing the biological activity of YA in the human body (Figure 1).

## 2. Materials and Methods

### 2.1. Chemicals

Tyr-Ala was prepared via protease hydrolysis of zein, which was catalyzed by the alkaline protease Alcalase, followed by isolation, purification, and synthesis by China Peptides Co., Ltd. (Shanghai, China). Streptozocin (STZ), D(+)-Sucrose, cholesterol, Sodium cholate, Propylthiouracil, 2′-7′dichlorofluorescin diacetate (DCFH-DA), and thiazolyl blue tetrazolium bromide ((3-(4,5-dimethylthiazol-2-yl))-2,5-diphenyltetrazolium bromide, MTT) were obtained from Sigma. Human amylin (hA) was purchased from Sunnyvale, which was used for preparing amylin stock solutions using hexafluoro-2-isopropanol (HFIP) as the solvent. The measured amounts of hA were solubilized in HFIP overnight to dissolve amylin completely. This approach efficiently removed preformed human amylin aggregates. Before the experiment, the HFIP solvent was evaporated with a gentle stream of nitrogen, and the peptide was then reconstituted in a reaction buffer to yield a final monomer concentration of 20 µM.

### 2.2. Cell Culture

The INS-1 cell line was derived from a rat islet cell tumor and cultured in RPMI-1640 medium (Gibco, New York, NY, USA) supplemented with 1 mM sodium pyruvate (Sigma, St. Louis, MO, USA), 50 µM β-mercaptoethanol (Sigma, St. Louis, MO, USA), 10% fetal bovine serum (Kangyuan Biology, Tianjin, China), and 1% penicillin/streptomycin (Solarbio, Beijing, China). The cells were maintained at 37 °C in a 5% CO_2_ atmosphere incubator and passaged every three days. The cells in the logarithmic phase of growth at passages of 6 to 20 were used to ensure reproducibility. After reaching 80% confluence, 1 × 10^4^ cells/well were seeded into 96-well plates for all experiments and maintained in a culture medium overnight unless otherwise specified.

### 2.3. MTT Reduction Assay

The reduction in MTT assay was used to assess the effect of YA on cell viability. The different concentrations of YA (10 µM, 20 µM, 40 µM) were added and co-incubated with H_2_O_2_ or hA for 24 h. MTT solution (1 mg/mL) was added to each well and incubated for 3 h at 37 °C. Next, MTT was replaced with isopropanol with 1% HCl, followed by shaking for 15 min. Colorimetric measurements of viable cell numbers were performed at 570 nm against a background measurement of 690 nm using a microplate spectrophotometer (Tecan Group AG, Männedorf, Switzerland) [20].

### 2.4. Reactive Oxygen Species (ROS) Assay

The DCFH-DA probe was used to assay the ROS in cells. The different concentrations of YA (10 µM, 20 µM, 40 µM) were added and co-incubated with H_2_O_2_ (50 μM). After treatment for 24 h, 10 mM DCFH-DA was added to each well and incubated for 30 min at 37 °C. Then, the cells were washed three times with HEPES buffer, and 150 µL/well HEPES buffer was added. The total fluorescence intensity was measured at an excitation wavelength of 480 nm and emission wavelength of 525 nm using a microplate spectrophotometer (Tecan Group AG, Männedorf, Switzerland) [21].

### 2.5. Insulin Secretion Assay

Insulin resistance model was established by incubating with 40 mM glucose for 24 h, while the administration group was added with different concentrations of YA (10 µM, 20 µM, 40 µM) and co-incubated with glucose. Then, cells were cultured for 1 h in a Krebs–Ringer buffer (KRB) containing 2.5 mM glucose and 16.7 mM glucose. Glucose-stimulated insulin secretion (GSIS) and basal insulin secretion (BIS) in the supernatant of cells were tested using an insulin secretion assay ELISA kit (R&D systems, Minnesota, USA). Then, the insulin levels represented by the insulin release index (GSIS/BIS) were calculated [22].

### 2.6. Western Blot

INS-1 cells were seeded into 6-well plates at 2 × 10^5^ cells/well overnight and then treated with glucose (40 mM) alone, co-incubation with glucose (40 mM) and YA (40 μM), YA (40 μM) alone for 24 h. Cells were washed twice with HEPES (4-(2-Hydroxyethyl)-1-piperazineethanesulfonic acid), and total lysates were prepared in cell lysis buffer (Cell Signal Technology Inc., Danvers, MA, USA). Equal proteins from cell extracts were separated by 12% SDS-PAGE and transferred onto a polyvinylidene difluoride membrane. The membrane was blocked with blocking buffer (136.7 mM NaCl, 2.68 mM KCl, 10.14 mM Na_2_HPO_4_, 1.76 mM KH_2_PO_4_ pH 7.5, 5% non-fat dry milk) at room temperature for 2 h. Then, the membranes were probed with primary antibodies (PI3K-P85 1:1000, Akt 1:1000, P-Akt (Ser 473) 1:1000, P-Akt (Thr 308) 1:1000, β-actin 1:1000) which were purchased by CST (Cell Signaling Technology, Inc., Boston, MA, USA), respectively, overnight at 4 °C. The blots were then incubated with corresponding horseradish peroxidase-conjugated secondary antibodies (HRP labeled goat anti-rabbit IgG antibody 1:1000, CST, Boston, MA, USA) for 1 h. Relative protein band intensities were detected using an enhanced chemiluminescence (ECL) reagent and quantified by the Quantity One software (Bioscience Biotech Co., Ltd., Shanghai, China).

### 2.7. Experimental Animals

**Figure 2:**
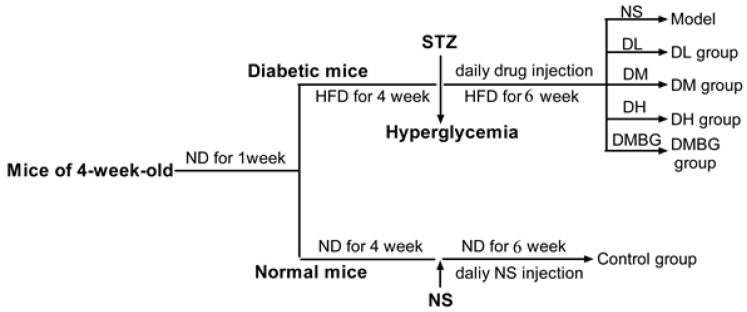
Schematic flow-chart of T2DM model mice establishment and experimental drug administration. T2DM mice were established by feeding HFD daily for 10 weeks and injecting STZ intraperitoneally at the fourth week. Starting from week 5, mice were administered DL, DM, DH, DMBG for 6 weeks. Each group contained 10 mice. (ND: control feed; HFD: high fat feed; STZ: streptozocin; NS: normal saline; DL: YA 5 mg/kg; DM: YA 10 mg/kg; DH: YA 20 mg/kg; DMBG: metformin).

Four-week-old male Chinese Kunming mice were purchased from the Experimental Animal Center of Jilin University. The mice had free access to food and water. After 1 week of acclimatization, the study commenced and lasted for 10 weeks (Figure 2). First, mice were divided randomly into two groups: the control group (*n* = 10), which received a normal diet, and the high-fat diet group (*n* = 50), fed a high-fat diet (20% D(+)-sucrose, 10% lard, 10% eggs, 1% cholesterol, 0.1% sodium cholate, 0.2% propylthiouracil, 58.7% normal diet). After 4 weeks of diet induction, the mice in the high-fat diet group were injected with 50 mg/kg of streptozocin (STZ) into the abdominal cavity for three days [23]. The fasting blood glucose of the high-fat diet group of mice, certified as successful T2DM of the model, was approximately higher than 11.1 mmol/mL. Then, T2DM model mice were randomly divided into the model (NS) group, YA (DL 5 mg/kg, DM 10 mg/kg, DH 20 mg/kg) group, and metformin (DMBG 100 mg/kg) group. After continuous tail vein injection of drugs, except control and model groups, for 6 weeks, the mice deprived of food overnight were sacrificed, and blood and tissue samples were collected for analysis. The experiments were conducted according to the Guidelines for the Care and Use of Laboratory Animals published by the United States National Institutes of Health (NIH Publication, revised 2011), and procedures were approved by the Animal Care and Drug Safety Evaluation committee of Jilin University.

### 2.8. Body Weight and Fasting Blood Glucose Assay

Body weight measurements were conducted weekly using an electronic scale in the morning. Fasting blood glucose was measured using a blood glucometer (GA-1, Sannuo Bio, Changsha, China) and test strips (Sannuo Bio). The first drop of blood was collected from the tip of the tail to minimize stress-induced changes in glucose levels.

### 2.9. Intraperitoneal Glucose Tolerance Test (IPGTT)

The anti-diabetic activity of dipeptide was measured in mice after injection of the drug for 4 weeks by IPGTT. After the mice were fasted overnight, 20% glucose (10 mg/kg body weight) in saline was intraperitoneally (IP) administered. Glucose levels were measured using a blood glucometer at 0, 30, 60, 90, and 120 min.

### 2.10. Biochemical Assay

Blood samples were withdrawn from the retro-orbital sinus and centrifuged (3500 rpm at 4 °C for 10 min). The serum was separated and stored at −20 °C for estimation of superoxide dismutase (SOD), malonaldehyde (MDA), glutathione (GSH), INS, hemoglobin A1C (HbA1c), total cholesterol (TC), and TG. INS, GSH, and MDA levels were measured using ELISA kits (Uscn Life Science Inc., Wuhan, China), while the others were measured using ELISA kits (Beyotime, Shanghai, China).

### 2.11. Histopathological Examination

At the end of the observational period, necropsy was performed on each animal to weigh the liver, spleen, pancreas, and kidneys and calculate the organ coefficient (viscera weight/body weight × 100%). The pancreas and kidneys were fixed in 10% buffered formalin and then processed via dehydration, embedding, sectioning at 5 m thickness, and hematoxylin and eosin (H&E) staining.

### 2.12. Statistical Analysis

Data were collected from several animals (*n* = 8/group) and presented as the mean ± SD. Comparisons were performed by one-way ANOVA for the different groups, followed by post hoc pairwise repetitive comparisons using Tukey’s test with GraphPad Prism software (GraphPad software, San Diego, CA, USA). Statistical significance was set at *p* < 0.05.

## 3. Results

### 3.1. Effect of YA on INS-1 Cell Viability

**Figure 3:**
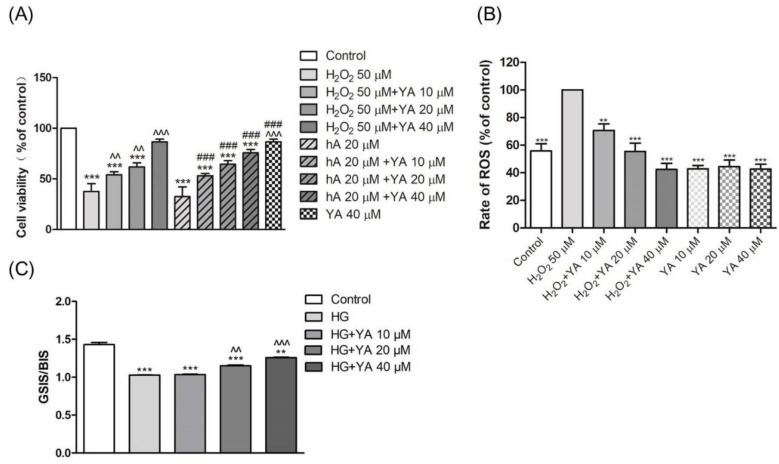
Antioxidant activity of YA in INS-1 cells. (**A**) The effect of YA on the toxicity of H_2_O_2_ and hA in INS-1 cells. INS-1 cells were treated with H_2_O_2_ (50 µM), co-incubation of H_2_O_2_ (50 µM) and YA (10, 20, and 40 µM), hA (20 µM), co-incubation of hA (20 µM) and YA (10, 20, and 40 µM), and YA (40 µM) alone for 24 h. Cell viability was measured using an MTT viability assay. (*** *p* < 0.001 vs. Control; ^^ *p* < 0.01 vs. H_2_O_2_; ^^^ *p* < 0.001 vs. H_2_O_2_; ^###^ *p* < 0.001 vs. hA); (**B**) Effect of YA on reactive oxygen species (ROS) accumulation in INS-1 cells. INS-1 cells were treated with H_2_O_2_ (50 µM) or YA (10 µM, 20 µM, 40 µM) for 24 h. The DCFH-DA method was used to assay the rate of ROS. (** *p* < 0.01 vs. H_2_O_2,_ *** *p* < 0.001 vs. H_2_O_2_); (**C**) Effect of different concentrations of YA on GSIS/BIS levels in INS-1 cells cultured by medium with 40 mM glucose. INS-1 cells were plated in a 96-well plate overnight and then treated with either glucose (40 µM) or co-incubation of glucose (40 µM) and YA (10 µM, 20 µM, 40 µM) for 24 h. (** *p* < 0.01 vs. glucose, *** *p* < 0.001 vs. glucose). All data are expressed as mean ± SD for each group (*n* = 3). HG: glucose 40 mM.

Cell viability was measured to assess the cytoprotective effect of YA on H_2_O_2^−^_ or hA-induced toxicity in INS-1 cells, as shown in Figure 3A. When INS-1 cells were treated with H_2_O_2_ (50 µM) or hA (20 µM), there was a decrease in cell viability. However, treatment of INS-1 cells with YA protected cells from H_2_O_2^−^_ or hA-induced toxicity. With the increase in YA concentrations, the INS-1 cell viability was increased.

### 3.2. Effect of YA on ROS Accumulation in INS-1 Cells

The DCF fluorescence intensity represents the ROS level. After treatment with H_2_O_2_ (50 µM), the green fluorescence intensity was obviously enhanced, which indicated that the level of intracellular ROS was increased (Figure 3B). The ROS levels of INS-1cells treated with co-treatment of YA (10 µM, 20 µM, and 40 µM) and H_2_O_2_ (50 µM) decreased compared to those of H_2_O_2_ alone. When INS-1 cells were treated with YA alone, the ROS levels were almost the same and lower than those in the control group. This indicates YA does not stimulate cell to produce ROS and perhaps reduces the ROS level in an islet tumor cell.

### 3.3. Effect of YA on Insulin Secretion

Secreted insulin levels were determined by GSIS/BIS. INS-1 cells were exposed to 2.5 mM glucose and 16.7 mM glucose in the KRB for 1 h, and GSIS/BIS were calculated. In contrast to the control, the treatment with 40 mM glucose for 24 h decreased the GSIS/BIS levels by almost 30% (Figure 3C). However, the GSIS/BIS levels increased when cells were treated with 20 or 40 µM YA (*p* < 0.01, *p* < 0.001).

### 3.4. Regulation of YA on PI3K/Akt Signal Pathway

**Figure 4:**
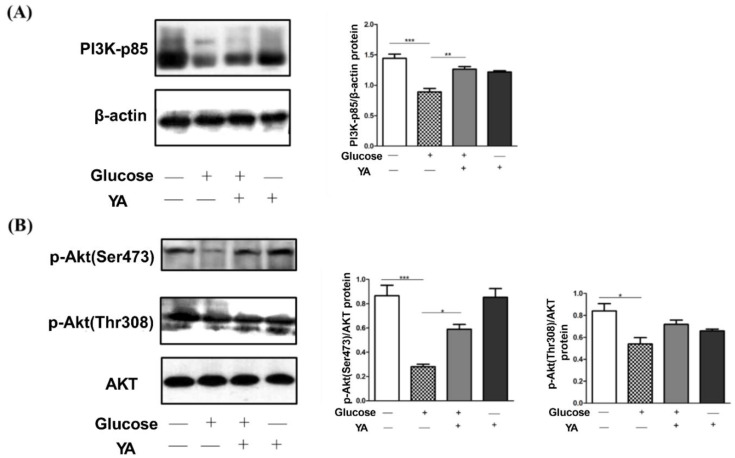
Effects of YA treatment on PI3K/Akt signaling pathway in INS-1 cells. INS-1 cells were treated with 40 μM YA and 40 mM glucose for 24 h, the PI3K (**A**) and phosphorylation of Akt473/Akt308 (**B**) were detected using Western blot. Equal amounts of proteins from each sample were separated on SDS-PAGE. Phosphorylation of Akt473/Akt308 was probed by p-Akt (Ser 473) and p-Akt (Thr 308) antibody. PI3K was probed by PI3K-p85 antibody. Total β-actin and Akt were taken as two control groups separately. Values are means ± SD from three representative experiments (*n* = 3). (* *p* < 0.05 vs. glucose, ** *p* < 0.01 vs. glucose, *** *p* < 0.001 vs. glucose).

As glucose promotes beta-cell proliferation via upregulation of the PI3K/AKT pathway, we firstly examined whether YA could stimulate PI3K-p85 production. Administration of 40 mM glucose significantly reduced PI3K-p85 protein levels compared to the control group, while co-incubation with 40 µM YA almost restored PI3K-p85 protein levels to untreated controls (Figure 4A). In addition, YA alone hardly affects PI3K-p85 protein levels, which are attributed to the stress-resistance effect by YA peptide [19]. Next, we investigated the effect of YA on p-Akt (Ser 473) and p-Akt (Thr 308) signaling. The levels of p-Akt (Ser 473) and p-Akt (Thr 308) in the glucose-treated group were significantly lower than those in the control group. However, after co-treatment with YA (40 µM), the levels of p-Akt (Ser 473) significantly increased, and p-Akt (Thr 308) rose a little (Figure 4B). Furthermore, the expression of p-Akt (Thr 308) of YA alone group reduced a bit. The results indicated that the effect of YA on inhibition of INS-1 injury induced by high glucose was more closely related to PI3K-p85 and p-Akt (Ser 473).

### 3.5. Effect of YA on General Condition and Weight of Mice

Mice in the control group appeared healthy, with lustrous fur and no hair loss. The intake of water and food did not obviously change. However, untreated T2DM mice were depressed and had mature gray fur. Water and food intake increased. After 6 weeks of YA treatment, the fur of the mice was brightening. Compared with untreated T2DM mice, the treated mice had reduced water and food intake.

**Figure 5:**
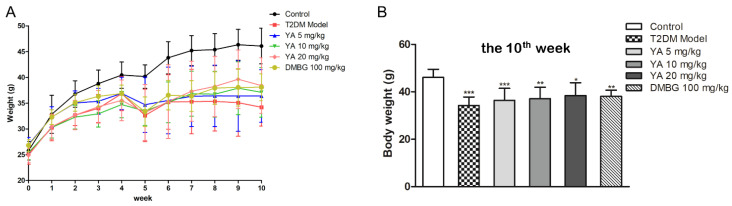
Effects of YA on body weight of T2DM mice. (**A**) Body weight differences between the different mouse groups. (**B**) Body weights of different groups mice on the 10th week. Data are expressed as mean ± SD for each group. (*n* = 8) (DMBG: metformin) (*** *p* < 0.001 vs. Control; ** *p* < 0.01 vs. Control; * *p* < 0.05 vs. Control).

Body weight is also an important indicator of mouse health. Compared with the control group, the weight gain of the mice in the T2DM group was slower. After treatment with different concentrations of YA and DMBG, the body weight was higher than that of T2DM mice after 5 weeks (Figure 5).

### 3.6. YA Decrease Blood Glucose Levels in T2DM Mice

**Figure 6:**
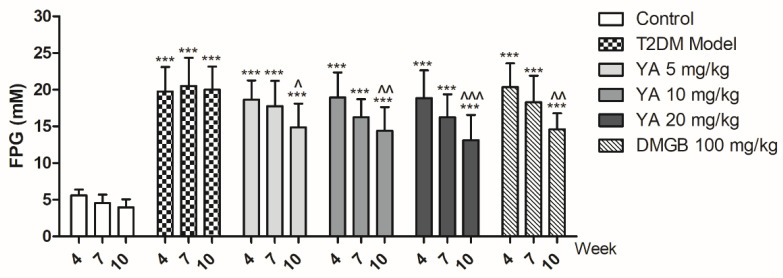
Fasting plasma glucose (FPG) of different groups during administration. Values are expressed as mean ± SD for each group. (*n* = 8) (*** *p* < 0.001 vs. Control group, ^ *p* < 0.05 vs. T2DM Model group at the 10th week, ^^ *p* < 0.01 vs. T2DM Model group at the 10th week, ^^^ *p* < 0.001 vs. T2DM Model group at the 10th week).

The fasting plasma glucose (FPG) levels of the T2DM mice increased in contrast to the normal mice and did not decline over time. FPG levels in the YA and DMBG groups decreased significantly compared to those in T2DM mice at the end of 10th week (Figure 6). It shows that YA can decrease glucose levels in T2DM mice during the continuous intravenous injection.

### 3.7. Effect of YA on Glucose Tolerance of T2DM Mice

**Figure 7:**
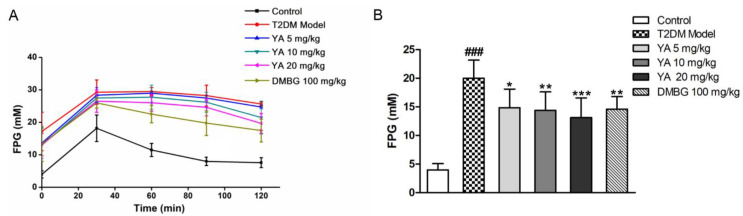
Effect of YA on FPG in different groups. (**A**) FPG of different groups at different time points; (**B**) FPG of different groups at 120 min. Data are expressed as mean ± SD for each group. (*n* = 8; DMBG: metformin) (### *p* < 0.001 vs. control, * *p* < 0.05 vs. T2DM model, ** *p* < 0.01 vs. T2DM model, *** *p* < 0.001 vs. T2DM model).

The glucose tolerance test showed the insulin sensitivity of T2DM mice. After 4 weeks of treatment, all groups of mice were challenged with a high glucose solution, and the FPG levels of mice reached a peak after 30 min. As time passed, FPG levels in the control mice decreased to normal levels within 120 min because of normal insulin secretion. However, the insulin sensitivity of the T2DM mice was weakened, and the FPG and blood glucose levels were higher than those in normal mice. During YA and DMBG treatment, FPG levels decreased much more rapidly than in untreated mice (Figure 7). This shows that YA improved insulin sensitivity in T2DM mice in a concentration-dependent manner.

### 3.8. Effect of YA on Insulin and HbA1c Levels

**Figure 8:**
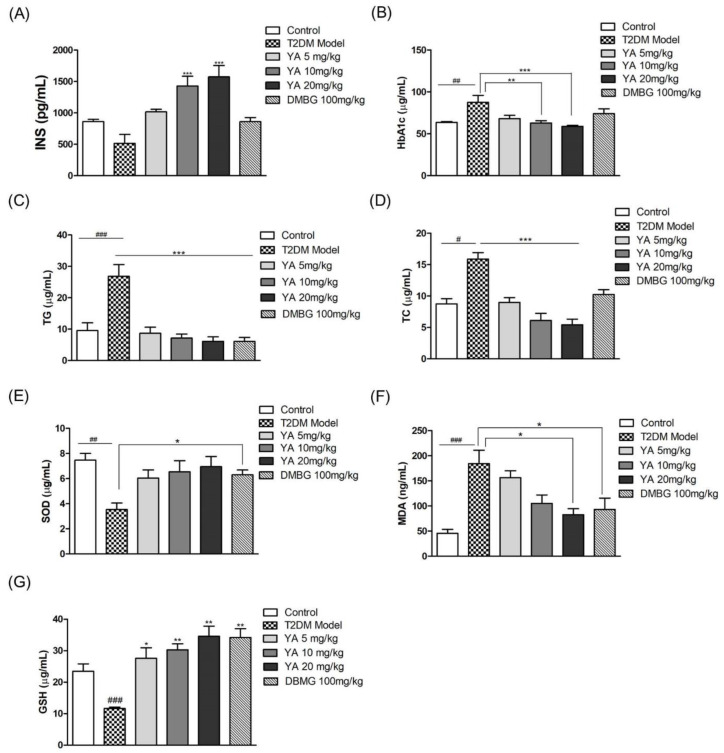
Effect of YA on the levels of insulin, hemoglobin A1C (HbA1c), triglyceride (TG), total cholesterol (TC), and malonaldehyde (MDA), and on the activities of superoxide dismutase (SOD) and glutathione (GSH) in serum. (**A**) insulin; (**B**) HbA1c; (**C**) TG; (**D**) TC; (**E**) SOD; (**F**) MDA; (**G**) GSH. Data are expressed as mean ± SD for each group. (*n* = 8; DMBG: metformin) (^#^ *p* < 0.05 vs. control, ^##^ *p* < 0.01 vs. control, ^###^ *p* < 0.001 vs. control, * *p* < 0.05 vs. model, ** *p* < 0.01 vs. model, *** *p* < 0.001 vs. model).

Compared with normal mice, the insulin levels of mice in the T2DM mice were decreased. Insulin levels in YA-treated mice increased in a concentration-dependent manner, and insulin levels in the medium-and high-dose YA groups were significantly higher than those in the T2DM mice and the control group. Compared with the T2DM model group, the insulin levels in the mice administered with DMBG increased, but the difference was not significant. The results demonstrated that YA could increase insulin levels in mice with type 2 diabetes (Figure 8A).

HbA1c is the product of the combination of hemoglobin and blood sugar, and its content is proportional to the blood sugar concentration. As shown in Figure 8B, the HbA1c levels in the T2DM model group had significantly increased. After administration of YA, the HbA1c levels were obviously decreased. Although HbA1c levels in the DMBG group were also reduced, there was no significant difference compared to the T2DM mice. In brief, YA had an inhibitory effect on HbA1c in T2DM mice, and the inhibitory ability was better than DMBG.

### 3.9. Effect of YA on TG and TC Levels

TG and TC levels in the T2DM mice were significantly higher than those in the control group. After YA and DMBG administration for 6 weeks, TG andTC levels were significantly decreased compared with those in the T2DM model group, and the high-dose YA and DMBG groups had slightly lower TG levels than the control group (Figure 8B–D). Overall, YA decreased TG and TC production in T2DM mice.

### 3.10. Effect of YA on Levels of SOD, MDA, and GSH

Long-term hyperglycemia could induce oxidative stress in vascular endothelial cells, and then, we measured redox indicators in mouse serum. SOD activity was much lower in the T2DM mice than in the control group. After the YA and DMBG treatment for 6 weeks, the SOD and GSH activities were effectively enhanced compared with untreated T2DM mice (Figure 8E,G). MDA levels in the T2DM mice were significantly higher than those in the control mice. Both the YA and DMBG groups had significantly suppressed MDA production in mice with type 2 diabetes. YA reduced MDA levels in mice in a concentration-dependent manner (Figure 8F). These results indicated that YA effectively alleviated oxidative stress in T2DM mice.

### 3.11. Effect on Liver, Spleen, Pancreas, and Kidney

**Figure 9:**
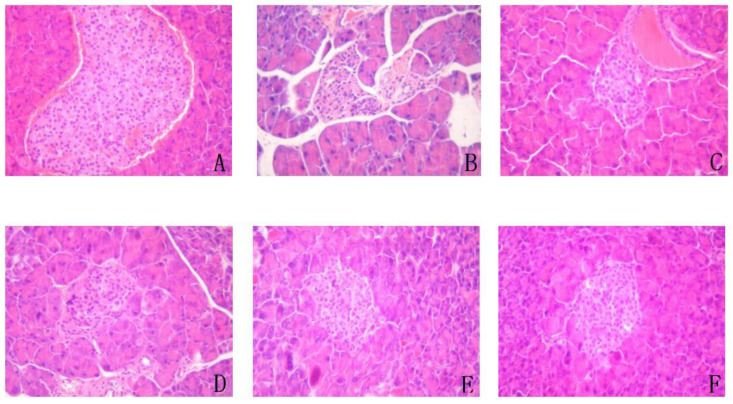
Representative hematoxylin and eosin (H&E) staining sections of pancreases from mice in the different groups (×200). (**A**). control; (**B**). T2DM mice; (**C**). YA 5 mg/mL; (**D**). YA 10 mg/mL; (**E**). YA 20 mg/mL; (**F**). DMBG 100 mg/mL.

**Figure 10:**
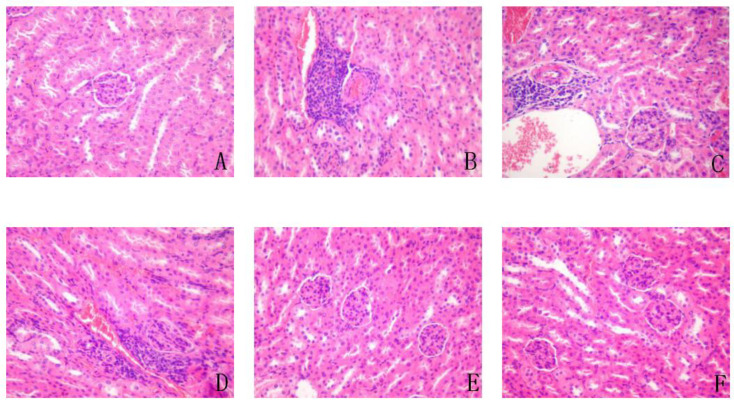
Representative H&E staining sections of kidneys from mice in the different groups (×200). (**A**). control; (**B**). T2DM mice; (**C**). YA 5 mg/mL; (**D**). YA 10 mg/mL; (**E**). YA 20 mg/mL; (**F**). DMBG 100 mg/mL.

The liver, spleen, pancreas, and kidneys of the T2DM mice were heavier than those of the control group, but the kidneys were most obviously increased. The weights of the organs from the YA and DMBG groups were lower than those of the T2DM mice (Table 1). The weight of organs in the YA-treated group decreased with increasing concentrations of YA, and the weight of spleen, pancreas, and kidneys were recovered to the same level as the control group when the YA concentration was 20 mg/kg. H&E staining of the pancreas and kidneys (Figure 9 and Figure 10) showed that the cytoplasm was dyed pink, and the nucleus was dyed blue. H&E staining of the pancreas (Figure 9) showed that the islets in normal mice were circular or elliptic funiculars with clear boundaries. These islet cells were well distributed with a good supply of blood vessels and tightly packed with several cytoplasm-rich cells. The nuclear boundaries of these cells were also clear. There were no obvious pathological changes in the pancreas. On the contrary, the pancreatic islets of T2DM mice were significantly reduced, smaller in size, sparsely arranged, and with blurred borders. The islet cells with nuclear pyknosis were swollen, necrotic, and with a visible vacuole degeneration. Unlike T2DM mice, after YA and DMBG treatment, the islet cells were relatively intact, with clear boundaries. The cells were found morphologically intact with no obvious swelling or necrosis. The nucleus pyknosis deformation also significantly decreased.

H&E staining of the kidneys (Figure 10) revealed clear cortical glomerular boundaries in the kidneys of control group mice, with clear and abundant glomerular stromal and renal tubular epithelial cells. There was no obvious inflammatory cell infiltration in interstitial renal cortical cells and no obvious pathological changes in glomeruli and tubules. In the T2DM mice, the number of adjacent glomerular stromal cells and nuclear enrichment was reduced, and the renal cortex was locally infiltrated with many inflammatory cells, mainly lymphocytes. Compared with T2DM mice, in mice treated with low and medium doses of YA, the renal cortex was not significantly changed; renal cortical interstitium was infiltrated by some inflammatory cells. In addition, the number of glomerular stromal cells decreased. After high-dose YA and DMBG treatment, the renal cortexes were relatively intact, and the glomerular boundaries were clear, with no obvious reduction in glomerular stromal cells. A few glomerular stromal cells were found to be pyknotic. These kidneys also had clear renal tubular epithelial cells and nucleoli. In addition, there was a few interstitial inflammatory cells in the renal cortex.

In conclusion, treatment with YA had a protective effect on the pancreas and kidneys of mice with type 2 diabetes.

## 4. Discussion

Pancreatic islet β-cell lines have different features; however, in this study, we used INS-1 cells, which contain more insulin secretion granules and produce fewer oxygen radical scavenging enzymes. Furthermore, INS-1 cells are negative for glucagon and amicine, thereby avoiding their influence on insulin secretion [24]. The number of islet β-cells changes with the severity of glucose metabolism disorders and insulin secretion [25]. Cell necrosis or apoptosis are important factors influencing the number of INS-1 cells, while apoptosis is the main driver of damage because of ROS oxidative stress [26]. Oxidative stress is induced in INS-1 cells by many factors, including high blood glucose, high concentrations of free fatty acids, streptozotocin (STZ), or H_2_O_2_. In normal physiological status, oxidative stress can activate antioxidant enzymes to reduce ROS-induced damage. However, low production of antioxidant enzymes in INS-1 cells leads to impaired ROS removal [27]. Excessive ROS can cause a decrease in the activity of INS-1 cells, ultimately leading to cell necrosis and apoptosis [28,29]. Insulin resistance is also related to oxidative-stress-induced dysfunction in type 2 diabetes [5]. In this experiment, we treated INS-1 cells with hydrogen peroxide, hA or high concentration of glucose to change ROS levels and establish an insulin resistance model. The results of YA treatment showed that YA could effectively improve cell viability and alleviate insulin resistance by removing ROS.

Islet amyloid (IA) deposition is a pathological feature of pancreatic islets in type 2 diabetes. Islet amyloid formation occurs through the aggregation of islet amyloid polypeptide (IAPP), which is normally secreted by beta cells [30]. Under normal physiological conditions, IA is secreted by β-cells and regulates blood glucose levels via insulin and glucagon. IA, except for insulin, is the only endocrine hormone that can lower blood glucose in vivo [31]. However, under pathological conditions, IA is a pathological product of T2DM. In the early stages of T2DM, the reduced secretory function of β-cells causes a decrease in insulin and amylin levels, which is an important mechanism, leading to IR and dysregulation of glucose metabolism [32]. Therefore, suppression of amylin is a feasible method for preventing and treating T2DM. This study showed that YA inhibits IAPP toxicity and improves cell viability.

Furthermore, researchers have found that suppression of the PI3K/Akt signaling pathway is implicated in high-glucose-induced cell dysfunction [33]. As shown in Figure 1, insulin activates and binds to the insulin receptor (InsR), inducing PI3K-AKT activation. The InsR is activated by insulin and binds to it, which in turn induces PI3K-AKT activation. After PI3K receives signals from tyrosine kinases or G-protein-coupled receptors, it recruits the regulatory subunit p85 to the plasma membrane and binds to the catalytic subunit p110, promoting phosphatidylinositol 4, 5-bisphosphate (PIP2) to generate phosphatidylinositol-3, 4, 5-triphosphate (PIP3) [34]. PIP3 binds to phosphoinositide-dependent kinase 1 (PDK1) through the signaling protein of the homology domain of AKT, induces the activated AKT to the cell membrane, and then the AKT is fully activated by phosphorylation of Thr308 and Ser473, regulating glycogen synthesis, gluconeogenesis, and glucose uptake [35]. Excessive ROS inhibits InsR phosphorylation and blocks downstream signaling pathways. In this study, YA reduced intracellular ROS levels and alleviated oxidative damage, and on the other hand, YA reversed the downregulation of Thr308/Ser473-phosphorylated Akt and PI3K-p85 induced by high glucose stimulation. These results indicated that YA reversed the effects of GSIS in INS-1 cells through the PI3K/Akt pathway.

T2DM is often associated with dyslipidemia, an initial factor in IR, and is a key component of many diseases, such as nonalcoholic fatty liver disease (NAFLD), hypertension, and hyperlipidemia [36,37]. Rising TG levels are usually a sign of dyslipidemia. Blood lipids include TC and TG, mostly from dietary intake, caused by consuming a high-fat and high-sugar diet, leading to the accelerated synthesis of TC and TG [38]. Thus, it is important to improve lipid metabolism in T2DM patients. In this study, during treatment with either YA or metformin for a month, both TC and TG levels in type 2 diabetic mice were significantly decreased. As treatment with YA reduced TC and TG levels, we inferred that YA might decrease blood lipids, reducing the complications of T2DM and arresting dyslipidemia.

FPG and HbA1c are important indicators for evaluating T2DM [39]. After treatment for 1 month, FPG levels in the YA and DMBG groups were significantly lower than those in the T2DM mice but were still higher than those in the control group. This indicated that YA can treat mice with type 2 diabetes; however, their blood glucose levels did not return to normal. HbA1c, formed by an irreversible metabolic reaction between hemoglobin and glucose, reflects the mean plasma glucose [40]. Our research indicated that during YA and DMBG treatment, FPG and HbA1c levels were markedly decreased compared with those in the T2DM mice, which could alleviate diabetes mellitus.

SOD, MDA, and GSH are the main biomarkers of oxidative stress [41]. SOD is an important antioxidant enzyme that can remove free radicals in the body, especially superoxide anions [42]. MDA, a degradation product of lipid peroxides and a toxic byproduct of lipid peroxidation in vivo, is relatively stable and easy to measure and is commonly used as an indicator of oxidative stress [43]. GSH mediates cellular redox homeostasis, protects the integrity of the cell membrane, and is an important indicator for measuring the antioxidant ability of an organism [44]. In this study, SOD and GSH levels were significantly increased, and MDA levels were significantly decreased in YA-treated T2DM mice. This could accelerate the TG/FFA cycle, which is likely to protect β-cells by avoiding excessive fuel and ROS production [11]. The results showed that YA increased GSH activity in type 2 diabetic mice, which was even higher than that in normal mice. Therefore, YA exhibits good oxidation resistance in vivo.

The animal viscera coefficient is an important measure for identifying genetic qualities of the animal, and the weight, shape, color, and luster of the viscera are important indicators of animal health. Lipid ectopic deposition refers to lipid accumulation in non-fatty tissues, such as the muscles and pancreas, but especially in the liver. If too many lipids accumulate in non-fatty tissues, it can cause serious damage to the organism. The liver, spleen, pancreas, and kidney viscera coefficients were higher in the model group than in the control group because of lipid accumulation. Furthermore, viscera coefficients of mice treated with YA or DMBG were decreased and approached the values seen in the normal mice. This indicates that YA may remove lipids from organs. Furthermore, H&E staining of the kidney and pancreas of mice showed that YA and DMBG treatment improved both the morphology and activity of islet cells, and the renal cortex also recovered. Furthermore, YA had a protective effect on the kidneys and pancreas of type 2 diabetic mice, resulting in a reduction in diabetes symptoms.

## 5. Conclusions

In conclusion, our study demonstrated that YA extracted from zein has a diabetes-relieving effect. YA attenuated oxidative damage to INS-1 cells by H_2_O_2_ or human amylin and promoted insulin secretion under high glucose stimulation in vitro. Moreover, YA treatment could reduce HbA1c, TC, TG, and MDA levels in T2DM mice, indicating that YA has a therapeutic effect on T2DM and its complications. This activity could be related to activate the PI3K/AKT signaling pathway with regulating oxidative stress, implying that YA may serve as a strategy to improve treatment of diabetes mellitus.

## Figures and Tables

**Table 1:** Weights of livers, spleens, pancreases, and kidneys in different groups. (# *p* < 0.05 vs. control; * *p* < 0.05 vs. T2DM model).

Group	Organs Coefficient ^@^			
	Liver	Spleen	Pancreas	Kidney
Control	3.95 ± 0.45	0.39 ± 0.06	0.56 ± 0.05	1.20 ± 0.26
T2DM Model	4.98 ± 0.58 *	0.50 ± 0.26	0.58 ± 0.09	1.55 ± 0.30
YA 5 mg/kg	4.75 ± 0.98	0.39 ± 0.15	0.53 ± 0.19	1.48 ± 0.21
YA 10 mg/kg	5.02 ± 0.51	0.41 ± 0.13	0.58 ± 0.16	1.65 ± 0.17
YA 20 mg/kg	4.75 ± 0.44 #	0.39 ± 0.17	0.53 ± 0.06	1.27 ± 0.61
DMBG 100 mg/kg	4.36 ± 0.51 #	0.26 ± 0.07	0.61 ± 0.14	1.54 ± 0.18

^@^ Values are expressed as mean ± SD for each group (*n* = 8).

## Data Availability

The data presented in this study are available in the article.
